# DNA methylation‐reprogrammed oxytocin receptor underlies insensitivity to oxytocin in pre‐eclamptic placental vasculature

**DOI:** 10.1111/jcmm.14299

**Published:** 2019-04-04

**Authors:** Xiaorong Fan, Ting Xu, Hongmei Ding, Huan Li, Yuxian Yang, Yun He, Jiaqi Tang, Yanping Liu, Xueyi Chen, Jie Chen, Jianying Tao, Zhice Xu, Qinqin Gao

**Affiliations:** ^1^ Institute for Fetology and Department of Obstetrics and Gynecology First Hospital of Soochow University Suzhou China; ^2^ Department of Obstetrics and Gynecology Affiliated Suzhou Hospital of Nanjing University of Chinese Medicine Suzhou China; ^3^ Department of Obstetrics and Gynecology Suzhou Municipal Hospital Suzhou China; ^4^ Center for Perinatal Biology Loma Linda University Sacramento California

**Keywords:** DNA methylation, oxytocin and oxytocin receptor, placental vascular dysfunction, pre‐eclampsia

## Abstract

Pre‐eclampsia is associated with inadequate placental blood flow and placental ischaemia. Placental vascular tone is essential for maintaining adequate placental blood flow. Oxytocin is increased in placental system at late pregnancy and onset of labour, and presented strongly concentration‐dependent contractions in placental vascular, suggesting that oxytocin could be involved in regulating placental vascular tone and circulation. However, information about the reactivity of oxytocin in pre‐eclamptic placental vasculature is limited. This study used a large number of human placentas to reveal the pathophysiological changes and its underlying mechanisms of oxytocin‐induced vasoconstrictions in placental vessels under pre‐eclamptic condition. Present study found that oxytocin‐induced contractions were significantly decreased in human pre‐eclamptic placental vasculature, associated with a deactivated transcription of *oxytocin receptor *gene. The deactivated *oxytocin receptor *gene transcription was ascribed to a relatively higher DNA methylation status of CpG islands in *oxytocin receptor* gene promoter. This study was first to reveal that a hyper‐methylation of CpG islands in *oxytocin receptor* gene promoter, leading to a relatively low pattern of oxytocin receptor expression, was responsible for the decreased sensitivity of oxytocin in pre‐eclamptic placental vessels.

## 1 INTRODUCTION

As the most common medical syndrome during human pregnancy, hypertensive disorders (especially pre‐eclampsia [PE]) are the leading causes of maternal and perinatal morbidity and mortality, and affect millions of women worldwide each year who had normal blood pressure before pregnancy.^1^, ^2^ Although the ultimate aetiology of PE is still unknown, placental ischaemia is a key in pathogenesis of PE which has been widely accepted.^3^, ^4^, ^5^ Placenta has two crucial roles namely: (a) transporting gases, nutrients and waste between maternal and foetal circulation systems; (b) immuno‐protection and releasing of chemicals necessary for pregnancy progress. It is undeniable that, as a new foeto‐maternal vascular organ, the maintenance of placental functions is dependent on sufficient placental perfusion and adequate blood flow via placental circulatory system.^6^ Generally, this system includes a placental vascular tree (composed of chorionic plate arteries and their branches, terminal villi and veins).^6^ Vascular tone in this system is essential for maintaining adequate placental blood flow. Because placental vessels lack autonomic innervation,^7^ local vasoconstrictors and vasodilators are of paramount importance for controlling blood flow in placental circulation. Abnormalities in constriction and relaxation of the placental vasculature will affect blood flow in the placenta, contributing to placental ischaemia and an abnormal course of pregnancy such as PE.^8^, ^9^ Therefore, the abnormal vascular reactivity of endogenous vaso‐activators in pre‐eclamptic placental vasculature offers interesting model for investigations of mechanisms as well as targets of treatments for PE. However, so far, there has been limited information on the abnormal vascular reactivity of endogenous vaso‐activators such as oxytocin (OXT) in pre‐eclamptic placental vasculature.

Oxytocin is a nine‐amino acid neuropeptide that is known to play a critical role in foetal expulsion and breastfeeding, and has been recently implicated in mammalian social behaviour.^10^, ^11^ The actions of both central and peripheral OXT are mediated through oxytocin receptor (OXTR), which is encoded by a single gene. In the context of pregnancy, OXT and OXTR are rich in the human placenta.^10^, ^12^ There is an increase in OXT concentrations in maternal plasma and the placenta with the onset of labour.^10^, ^11^ As a classical vasoconstrictor, OXT is predominantly known as a potent stimulator of uterine smooth muscle, and studies on isolated placental vessels have suggested an important role for this compound in the maintenance of placental vascular tone and circulation.^13^ However, information about the reactivity of OXT in pre‐eclamptic placental vasculature is limited. This was the first study using a large number of human placental vessels, to reveal the pathophysiological changes and its underlying mechanisms of OXT‐induced vasoconstrictions in placental vessels under the pre‐eclamptic condition, which would provide new information for better understanding the pre‐eclamptic placental vascular dysfunctions and placental ischaemia.

## 2 MATERIALS AND METHODS

### 2.1 Patients and samples

Placenta specimens of normal (NP, N = 45) or pre‐eclamptic pregnancy (PE, N = 42) were immediately collected after vaginal delivery from the local hospitals, Suzhou, China. The study protocols were approved by the Ethics Committee of First Hospital of Soochow University (ref. no. 2015‐128). Written informed consent was obtained from all enroled patients in accordance with the Declaration of Helsinki (2013) of the World Medical Association. Normal pregnancies were defined as those with blood pressure < 120/90 mm Hg with no significant complications. Pre‐eclamptic pregnancies were defined by the onset of hypertension during pregnancy (blood pressure ≥ 140/90 mm Hg, with no prior hypertension history) and consistent proteinuria (≥300 mg/d).^1^, ^14^ The clinical characteristics of all participants were summarized in Table 1.

**Table 1:** Basic characteristics of pre‐eclampsia cases and normotensive controls

Characteristics	NP	PE
Number of participants	45	42
Maternal age (y)	28.1 ± 4.6	28.3 ± 4.2
Gestational age (wk)	38.1 ± 2.1	33.5 ± 4.4**
Birth weight (kg)	3.3 ± 0.7	2.6 ± 0.9*
Systolic BP (mm Hg)	111.4 ± 7.8	154.3 ± 19.6**
Diastolic BP (mm Hg)	79.5 ± 9.6	109.2 ± 12.9**
Proteinuria (g/24 h)	0.16 ± 0.07	5.21 ± 2.58**
S/D ratio	2.02 ± 0.41	3.816 ± 1.86*

The data were expressed as mean ± SD. Pre‐eclampsia vs. normal pregnant. S/D ratio, ratio of systolic and diastolic blood flow in the umbilical artery. PE, pre‐eclampsia; NP, normal pregnant.

* *P* < 0.05.

** *P* < 0.01.

### 2.2 Vascular functional studies

Placental tissue were kept in iced Krebs solution (containing in mmol/L: NaCl 119, NaHCO3 25, glucose 11, KCl 4.7, KH2PO4 1.2, MgSO4 1.0 and CaCl2 2.5), and bubbled with 95% O2 and 5% CO2. Human placental vessels (HPV) (HPV‐A1/A2, first‐, second‐order branch of blood vessels in the placenta, mainly the main stem villous arteries; HPV‐A3, branch of the main stem villous arteries with diameter around 100 m) were carefully isolated. HPV were cut into rings approximately 3‐5 mm in length and mounted in a multi‐myograph system for recording of isometric tension (PowerLab 16/SP and Chart 5).^15^ Vessel rings were adjusted to maintain a suitable passive force and equilibrated for 60 minutes. Potassium chloride (KCl, 120 mmol/L) was used to achieve optimal resting tension before adding drugs. The contractions elicited by KCl were a reference for contractile capacity in response to drugs.^16^ The vessel rings were contracted with cumulative concentrations of OXT (10^−10^ to 10^−4^ mol/L in HPV‐A1/A2; 10^−8^ to 10^−4 ^in HPV‐A3). Atosiban (10^−5^ mol/L) was used for pre‐treating segments for 30‐60 minutes before application of OXT. All drugs were purchased from Sigma‐Aldrich (Saint Louis, MO, USA).

### 2.3 DNA isolation and targeted bisulphite sequencing assay

To prepare genomic DNA, homogeneous HPV were lysed with the lysis buffer containing (10 mmol/L Tris‐Cl (pH 7.5), 10 mmol/L NaCl, 10 mmol/L EDTA, 0.5% sarcosyl, and 1 mg/ml proteinase K), and incubated overnight at 60°C. Genomic DNA was extracted from the lysates by standard phenol/chloroform technique and subjected to bisulphite conversion using EZ DNA Methylation^TM^‐GOLD Kit (Zymo Research) according to manufacturer's protocols. Sodium bisulphite preferentially deaminates unmethylated cytosine residues to thymines, whereas methyl‐cytosines remain unmodified. DNA was quantified and then diluted to a working concentration of 20ng/μL for quantitative methylation analysis. BiSulfite Amplicon Sequencing (BSAS) was used for quantitative methylation analysis.^17^ CpG islands located in the proximal promoter of OXTR were selected for measurement according to the following criteria: (a) ≥200 bp length; (b) ≥50% GC content; (c) ≥60% ratio of observed/expected CpG dinucleotides.^18^ Based on the genomic coordinates of the 22 candidate CpG sites of *OXTR*, we carefully designed the primers in order to detect them in a panel (Table 2). After PCR amplification, products were sequenced by Illumina Hiseq 2000. Each tested CpG site was named according to its relative distance (in bp) to transcriptional start site (TSS). Methylation level at each CpG site was calculated as the percentage of the methylated cytosines over the total tested cytosines. The average methylation level was calculated using methylation levels of all measured CpG sites within *OXTR* gene.

**Table 2:** Methylated CpG sites measured in this study

Gene	Position	Genomic location	Relative to TSS, bp
*OXTR*	1	Chr3: 8811075	+225
	2	Chr3: 8811090	+210
	3	Chr3: 8811093	+207
	4	Chr3: 8811108	+192
	5	Chr3: 8811128	+172
	6	Chr3: 8811132	+168
	7	Chr3: 8811139	+161
	8	Chr3: 8811153	+147
	9	Chr3: 8811155	+145
	10	Chr3: 8811157	+143
	11	Chr3: 8811159	+141
	12	Chr3: 8811166	+134
	13	Chr3: 8811176	+124
	14	Chr3: 8811179	+121
	15	Chr3: 8811186	+114
	16	Chr3: 8811209	+91
	17	Chr3: 8811213	+87
	18	Chr3: 8811219	+81
	19	Chr3: 8811229	+71
	20	Chr3: 8811233	+67
	21	Chr3: 8811243	+57
	22	Chr3: 8811245	+55

### 2.4 Quantitative real‐time PCR and Western blot analysis

Total RNA was isolated from placental vessels using Trizol reagent, and was reversed transcribed using the first‐strand cDNA Synthesis Kit (Invitrogen Corp., Carlsbad, CA, USA). Then, qRT‐PCR was performed with the SYBR Green Supermix Taq Kit (Takara Biotechnology Co., Ltd., Dalian, China) and analysed on an iQ5 Real‐Time PCR Detection System (Bio‐Rad Laboratories, Inc, Hercules, CA, USA). ∆∆Ct method was used to comparatively quantify the abundance of mRNA levels. The qRT‐PCR primer sequences were listed in Table 3. The protein abundance of OXTR in placental vessels was assessed by Western blot analyses normalized to β‐actin. Antibodies were purchased from Sigma‐Aldrich or CST (Cell Signaling Technology, Inc China). All experiments were repeated three times with independently prepared tissue, and performed as previously described.^19^

**Table 3:** The primers used in this study

Primer	Nucleotide Sequence (5'‐3')
qRT‐PCR primers	
OXTR	Sense: TCAGCAGCGTCAAGCTCATC
	Anti‐sense: GTGAACAGCATGTAGATCCAG
Bisulphite sequencing primers	
OXTR	Sense: TTTYGTTTYGGAGGGGTTTG
	Anti‐sense: AATACTAAACTAAAATCTCTCACTAAAACCTC

### 2.5 Data analysis and statistics

Data are presented as mean ± SEM, and analysed by a two‐tailed Student's *t* test or two‐way analysis of variance (ANOVA) followed by Bonferroni test using GraphPad Prism version 5 (GraphPad Software, San Diego, CA, USA), where appropriate. DNA methylation/mRNA correlation plot for *OXTR* gene was identified by causal inference test (SigmaPlot 10.0). Results were considered statistically significant when the *P* value was less than 0.05.

## 3 RESULTS

### 3.1 Oxytocin‐induced contractions in human placental vessels

**Figure 1:**
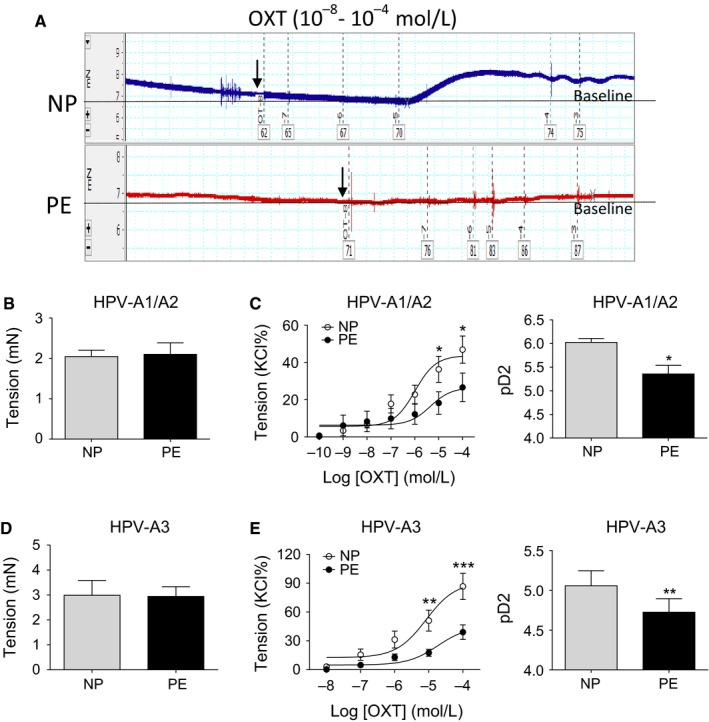
OXT‐mediated vascular reactivity in human placental vessels. A, C and E, Concentration‐response curves of OXT‐induced contractions in HPV‐A1/A2 (N = 18, n = 36) and HPV‐A3 (N = 18, n = 36). B and D, KCl‐induced maximal contractions in HPV‐A1/A2 and HPV‐A3 (N = 23, n = 35). KCl, potassium chloride; NP, normal pregnancy; PE, pre‐eclampsia; HPV‐A1/A2, first‐, second‐order branch of placental vessels; HPV‐A3, the third branch of placental vessels (micro‐vessels with diameter around 100 m). Error bars denote sem *P < 0.05; **P < 0.01; ***P < 0.001. N, number of participants; n, number of rings

In both HPV‐A1/A2 and HPV‐A3, there were no significant differences in KCl‐induced maximal contractions between normal NP and PE group (Figure 1B and D). Whereas, in both of HPV‐A1/A2 and HPV‐A3 from PE group, OXT‐induced contractions were significantly decreased with a reduced maximal response and pD2 (−log [50% effective concentration]) as compared to NP group (Figure 1A, C and E, *P* < 0.05). These data indicated that pre‐eclamptic placental vessels were significantly insensitive to OXT.

### 3.2 Expression of OXTR in human placental vessels

**Figure 2:**
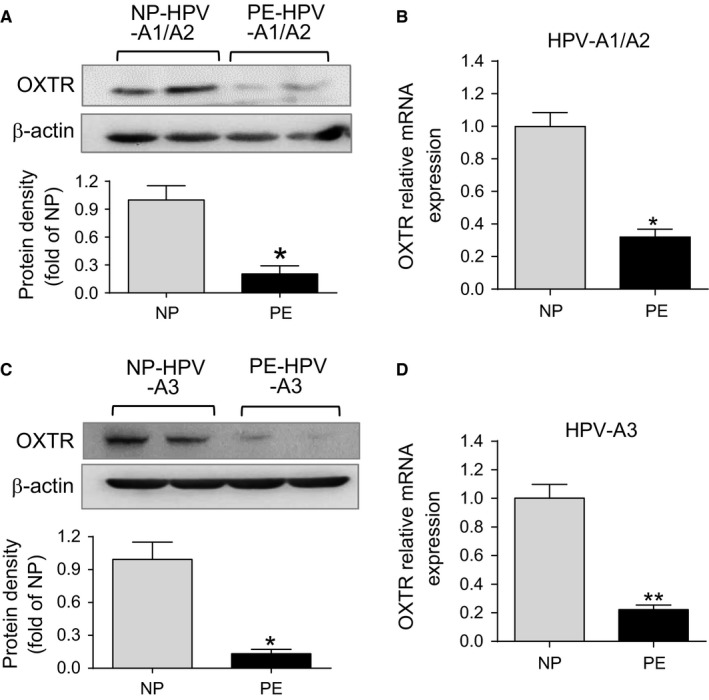
Expression of OXTR in human placental vessels. A‐D, The protein and mRNA levels of OXTR in HPV‐A1/A2 and HPV‐A3 were determined by Western blot analyses and qRT‐PCR (N = 15, n = 30 each group). Error bars denote sem *P < 0.05; **P < 0.01. N, number of participants

To investigate whether the decreased OXT‐mediated vasoconstrictions were correlated with the altered OXTR expression in pre‐eclamptic placental vessels, OXTR mRNA and protein levels were determined. As shown in Figure 2A‐C, there was a significant decrease in protein of OXTR in both of HPV‐A1/A2 and HPV‐A3 from PE group. To determine whether OXTR expression was regulated through transcriptional mechanism, we measured the mRNA abundance of OXTR. Figure 2B‐D showed that compared with NP group, the mRNA levels of OXTR in HPV‐A1/A2 and HPV‐A3 were decreased in PE group. These data suggested that the decreased sensitivity to OXT in pre‐eclamptic placental vessels was correlated with the down‐regulated OXTR, and the deactivated transcription of *OXTR *gene.

### 3.3 The effect of atosiban on OXT‐induced contractions in human placental vessels

**Figure 3:**
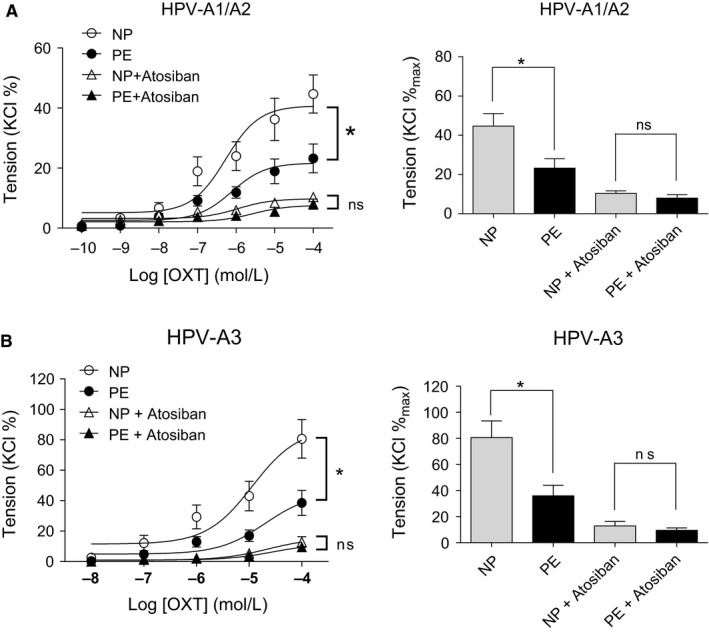
The effect of atosiban on OXT‐induced contractions in human placental vessels. A and B, The inhibitory effect of atosiban on OXT‐induced contractions in HPV‐A1/A2 (N = 12, n = 25) and HPV‐A3 (N = 10, n = 16). Atosiban, an OXTR antagonist. Error bars denote sem *P < 0.05; **P < 0.01; ***P < 0.001; ns, no significances. N, number of participants; n, number of rings

To further determine whether the decreased OXTR was associated with OXT‐induced vasoconstrictions in PE group, vascular rings were pre‐treated with atosiban (OXTR‐specific antagonist). As shown in Figure 3, atosiban almost completely blocked OXT‐mediated contractions in both of HPV‐A1/A2 and HPV‐A3, without differences between NP and PE groups after pre‐treated with atosiban, demonstrating that the decreased OXT‐mediated vasoconstrictions in PE group were due to the down‐regulated OXTR.

### 3.4 DNA methylation of CpG locus at *OXTR* gene promoter in human placental vessels

**Figure 4:**
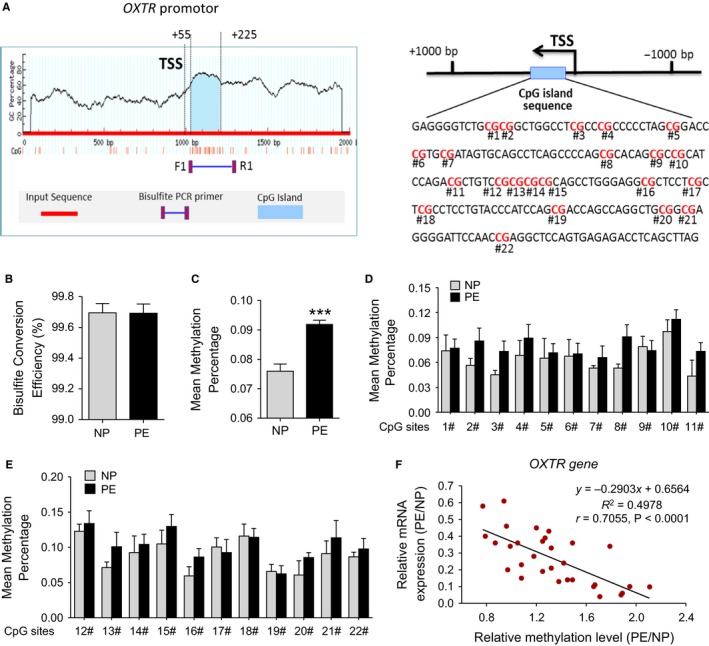
DNA methylation of CpG locus at *OXTR* gene promoter in human placental vessels. A, Bioinformatic analysis of the CpG islands of the *OXTR* gene from upstream −1 kb to downstream +1 kb region. Sequence analysis identified one CpG island in exon 1 that contains 22 CpG sites, located at positions +55 to +225 from the translation start site (TSS, defined as position 1) in the *OXTR* gene promoter. B, Represent the bisulphite conversion efficiency between NP and PE group. Bisulphite conversion efficiency was calculated by the number of transformed C to T divided by the number of C in each sample. C‐E, Represent the mean methylation status of the genomic regions in *OXTR* gene promoter. Each point represents mean methylation percentage in a genomic region of a sample. F, Expression analysis of *OXTR* gene and its correlation with methylation levels of 22 CpG sites. DNA methylation/mRNA correlation plots for *OXTR* gene identified by causal inference test. The y‐axis represents the relative expression level of *OXTR* gene which was detected with qRT‐PCR method. The x‐axis represents the relative mean methylation level of all the 22 CpG sites within *OXTR* gene. r: Pearson correlation coefficient. Error bars denote sem ***P < 0.001. N, number of participants

The expression of a gene may be down‐regulated through an increase in DNA methylation of its promoter, CpG islands.^18^ Sequence analysis identified one CpG island that contains 22 CpG sites within exons of the *OXTR* gene (Figure 4A). *OXTR* located on chromosome 3p25. Table 2 provides a key for CpG labels. We validated methylation status for the 22 CpG sites with targeted bisulphite sequencing. The bisulphite conversion rate of each sample was higher than 99%, and no significant differences were found between NP and PE group, indicating the bisulphite conversion was efficient and reliable in the experiments (Figure 4B). By using targeted bisulphite sequencing that examined in vivo methylation pattern of CpG island region, we found that, compared to NP, mean methylation percentage of the total 22 CpG sites within *OXTR* gene promoter in PE group was remarkably increased (PE vs NP: 0.0760 ± 0.0022 vs 0.0919 ± 0.0014, *P* < 0.001; Figure 4C), whereas, no significant difference was found in each tested CpG site between NP and PE group (Figure 4D‐E). Position and methylation levels of the 22 CpG sites in HPV were listed in Tables 2 and 4. Correlation analysis between *OXTR* gene methylation and expression was also conducted. As shown in Figure 4F, there was a significantly inverse correlation between the methylation status of 22 CpG sites in *OXTR* gene promoter and *OXTR* gene expression.

**Table 4:** Methylation levels of 22 CpG sites in OXTR gene promoter

Position	NP	PE
1	0.074 ± 0.019	0.077 ± 0.011
2	0.057 ± 0.008	0.086 ± 0.015
3	0.045 ± 0.005	0.073 ± 0.012
4	0.069 ± 0.018	0.089 ± 0.016
5	0.065 ± 0.024	0.072 ± 0.011
6	0.068 ± 0.020	0.070 ± 0.013
7	0.053 ± 0.003	0.066 ± 0.014
8	0.053 ± 0.005	0.091 ± 0.014
9	0.079 ± 0.012	0.074 ± 0.012
10	0.097 ± 0.014	0.112 ± 0.011
11	0.044 ± 0.019	0.073 ± 0.010
12	0.123 ± 0.010	0.134 ± 0.018
13	0.071 ± 0.008	0.101 ± 0.020
14	0.093 ± 0.024	0.104 ± 0.014
15	0.105 ± 0.020	0.130 ± 0.017
16	0.059 ± 0.013	0.086 ± 0.012
17	0.100 ± 0.013	0.093 ± 0.018
18	0.116 ± 0.017	0.114 ± 0.013
19	0.066 ± 0.010	0.062 ± 0.011
20	0.061 ± 0.020	0.086 ± 0.007
21	0.091 ± 0.018	0.114 ± 0.025
22	0.087 ± 0.007	0.098 ± 0.015
Average	0.0760 ± 0.0022	0.0919 ± 0.0014***

NP, normal pregnancy; PE, pre‐eclampsia; Error bars denote SEM.

*** *P* < 0.001.

## 4 DISCUSSION

This study found that OXT‐induced vascular contractions were significantly decreased in pre‐eclamptic placental vasculature. The decreased sensitivity to OXT in pre‐eclamptic vessels was correlated with the down‐regulated OXTR, as well as the deactivated transcription of *OXTR* gene. *OXTR* gene transcription was linked with DNA methylation status of CpG islands in *OXTR* gene promoter. To the best of our knowledge, this is the first study to reveal that a hyper‐methylation of certain CpG islands in *OXTR *gene promoter led to a relatively reduced pattern of OXTR expression, which was responsible for the decreased sensitivity to OXT in placental vessels under pre‐eclamptic conditions. Thus, the data not only offered new information for further understanding the pathological features and mechanisms of PE, but also underlined a role of epigenetic‐mediated gene expression in pre‐eclamptic placental vascular dysfunctions such as placental ischaemia.

In uterus, large (HPV‐A1/A2) and small (HPV‐A3) vascular branches in the placenta play a key role in maintaining a balance of placental circulation and provide adequate blood flow for development of foetus. Present data showed that in both pre‐eclamptic large and small placental vessels, OXT‐induced vascular contractions were significantly decreased with a reduced maximal response and pD2 as compared to normal pregnancy, indicating that pre‐eclamptic placental vessels were significantly insensitive to OXT. To date, information about the reactivity of OXT in pre‐eclamptic placental vessels was limited. One study in 1989 reported that OXT produced weak contractions in placental vessels, and no significantly differences in OXT‐induced contractions between normal and pre‐eclamptic placental vessels.^20^ Based on these results, previous study suggested that the effects of OXT on placental vascular tone in human term pregnancy are negligible. However, considering huge variation in human participants and the sample size was small (only six per group), it is hard to confirm OXT effects on human placental vessels. The similar situations were noted regarding vascular effects of acetylcholine on placental vessels recently, as obvious controversy results existed before.^8^, ^16^, ^21^, ^22^ To solve the problem, we significantly increased sample size in order to reach repeatable results.^15^, ^16^ In the present study, the large number of human samples (60 independent vessel tests, more than 40 placentas per group) was used for functional analysis. At least 85% in 60 independent tests showed OXT‐induced contractions were significantly decreased in pre‐eclamptic placental vessels.

Oxytocin exerts its effects on cells through interaction with OXTR, a G‐protein coupled receptor that upon ligand binding transduces signal to the nucleus. To investigate whether OXTR mediates OXT‐decreased vasoconstrictions in pre‐eclamptic placental vessels, placental vessel rings were pre‐treated with atosiban (an OXTR‐specific antagonist). Atosiban almost completely inhibited OXT‐induced contractions in placental vessels, without significant differences in OXT‐induced contractions between normal and pre‐eclamptic placental vessels after pre‐treatment with atosiban. These results indicated that OXTR mediated OXT‐decreased vasoconstrictions in pre‐eclamptic placental vessels. Furthermore, the protein levels of OXTR were decreased in pre‐eclamptic placental vessels. Interestingly, the mRNA abundance of OXTR was also significantly decreased in pre‐eclamptic placental vessels. These data demonstrated that the decreased OXT‐mediated vasoconstrictions in pre‐eclamptic placental vessels were correlated with the deactivated transcription of OXTR.

Numerous studies reported that transcription of OXTR was found to be partly regulated by epigenetic mechanisms in animal and human tissues.^23^, ^24^, ^25^, ^26^, ^27^ DNA methylation is a major epigenetic mechanism that regulates gene transcription and there is a strong correlation between gene expression and DNA methylation in gene promoter.^18^ Hyper‐methylation of CpG sites in the promoter of genes is often associated with suppression of transcription.^18^ A number of studies in humans and laboratory animals indicated that the methylation of CpG islands in *OXTR* gene promoter suppressed its transcription and may regulate tissue‐specific gene expression in organs.^23^, ^24^, ^25^ Our sequence analysis identified one CpG island that contains 22 CpG sites in *OXTR* gene promoter. To determine possible epigenetic dysregulation of *OXTR* gene, DNA methylation status of the 22 CpG sites was evaluated. Although no differences were found in each tested CpG site, the total mean methylation level of 22 CpG sites in pre‐eclamptic placental vasculature was significantly increased. Next, we conducted the causal inference test to test *OXTR* gene expression and its correlation with methylation levels of the CpG island, and found a significantly inverse correlation between the methylation status of CpG island in *OXTR* gene promoter and its transcription. In line with previous studies in the brain and peripheral tissue,^23^, ^24^ the deactivated *OXTR* transcription were also linked with reprogrammed DNA methylation patterns in pre‐eclamptic placental vessels. This study was the first to demonstrate a hyper‐methylation of CpG sites in the promoter of *OXTR* gene in pre‐eclamptic placental vessels, which was responsible for the decreased OXTR expression, and eventually led to insensitivity to OXT. In the myometrial and placental tissue of humans, OXTR was shown to be rise as pregnancy progress and to be maximally expressed after labour onset.^28^, ^29^ This expression pattern is consistent with PE onset time, which usually happens after the 20th week of gestation. This association raised the possibility that OXT/OXTR system may play a unique role in the regulation of placental vascular tone, and its abnormalities may be involved in the pathogenesis of PE. Thus, this study offered new information on specific pathological features of PE, and suggested there is a role for OXT/OXTR system in placental vascular dysfunctions for better understanding the pathogenesis of PE.

The present study found a special pattern of OXT‐mediated vascular contractions in pre‐eclamptic placental vasculature. The decreased sensitivity to OXT in pre‐eclamptic vessels was correlated with the down‐regulated OXTR and deactivated transcription of *OXTR*, which was linked to a hyper‐methylation of CpG islands in *OXTR* gene promoter. Significances of the finding include: (a) offering new information for further understanding the pathological features and mechanisms of PE; (b) underlining a role of epigenetic‐mediated gene expression in pre‐eclamptic placental vascular dysfunctions and placental ischaemia state. In addition, as an uterotonic agent to facilitate childbirth and reduce postpartum bleeding, OXT has been used widely in clinical practice. The finding that ‘placental vessels in PE showed a relative insensitivity pattern to OXT’ implied possibilities for individual treatments as well as precision medicine for using OXT. Whether OXT doses need to be differentiated between the normal and pre‐eclamptic women is worth pondering and discussing.

## CONFLICT OF INTEREST

The authors have none to declare.

## AUTHOR CONTRIBUTIONS

QG processed the data and figures, and performed vessel experiments with FX, TX, HD, YY, YH, JT, YL, XC and HL. FX and JC processed the data and performed molecular studies. JT and ZX prepared human umbilical cord samples. The work was supervised by ZX and QG.
